# Evaluating the Oxidative Stress in Inflammation: Role of Melatonin

**DOI:** 10.3390/ijms160816981

**Published:** 2015-07-27

**Authors:** Aroha Sánchez, Ana Cristina Calpena, Beatriz Clares

**Affiliations:** 1 Department of Pharmacy and Pharmaceutical Technology, School of Pharmacy, University of Barcelona, Joan XXIII Avenue, Barcelona 08028, Spain; E-Mails: asanchmi12@alumnes.ub.edu (A.S.); anacalpena@ub.edu (A.C.C.); 2 Department of Pharmacy and Pharmaceutical Technology, School of Pharmacy, University of Granada, Campus of Cartuja Street, Granada 18071, Spain

**Keywords:** antioxidant, melatonin, oxidative stress, inflammation, chronic diseases

## Abstract

Oxygen is used by eukaryotic cells for metabolic transformations and energy production in mitochondria. Under physiological conditions, there is a constant endogenous production of intermediates of reactive oxygen (ROI) and nitrogen species (RNI) that interact as signaling molecules in physiological mechanisms. When these species are not eliminated by antioxidants or are produced in excess, oxidative stress arises. Oxidative stress can damage proteins, lipids, DNA, and organelles. It is a process directly linked to inflammation; in fact, inflammatory cells secrete a large number of cytokines and chemokines responsible for the production of ROI and RNI in phagocytic and nonphagocytic cells through the activation of protein kinases signaling. Currently, there is a wide variety of diseases capable of producing inflammatory manifestations. While, in the short term, most of these diseases are not fatal they have a major impact on life quality. Since there is a direct relationship between chronic inflammation and many emerging disorders like cancer, oral diseases, kidney diseases, fibromyalgia, gastrointestinal chronic diseases or rheumatics diseases, the aim of this review is to describe the use and role of melatonin, a hormone secreted by the pineal gland, that works directly and indirectly as a free radical scavenger, like a potent antioxidant.

## 1. Introduction

Eukaryotic cells use the oxidation of nutrients as an energy source to obtain ATP. In the complete oxidation process of glucose by cells, several metabolic pathways (glycolysis, Krebs cycle, and electron transport chain (ETC)) and several cell compartments (cytoplasm, membrane and mitochondrial matrix) are involved. In the ETC, electrons from nicotinamide–adenine–dinucleotide phosphate (NADH) are transported sequentially by a series of redox reactions through the complex I, ubiquinone, complex III cytochrome c, and finally to the complex IV, where in a reaction catalyzed by cytochrome oxidase, electrons are sold to molecular oxygen producing H_2_O. Thus, the major role of oxygen for all aerobic organisms is acting as final acceptor for electrons [1]. Under physiological conditions, enzymes of the mitochondrial respiratory chain, NADPH, succinate dehydrogenase (SDH), ubiquinone, cytochrome c reductase, cytochrome b5, monoamine oxidases (MAO-A and MAO-B), mitochondrial aconitase, ketoglutarate dehydrogenase complex (KGDHC, 2-oxoglutarate dehydrogenase), dihydroorotate dehydrogenase (DHOH), pyruvate dehydrogenase complex (PGDH), oxoglutarate dehydrogenase complex (OGDC), as a consequence of metabolism, are responsible for a constant endogenous production of free radicals (FR) as reactive oxygen intermediates (ROI) and nitrogen species (RNI) [2]. These intermediates interact as signaling molecules for metabolism, cell cycle, and intracellular transduction pathways [3]. These species are also produced in response to exposure to exogenous agents as UV, ionizing radiation or air pollution [4]. The main source of reactive oxygen species (ROS) generation is the ETC in mitochondria. However, there are also non-mitochondrial sources of reactive molecules and FR that include respiratory burst of phagocytic cells, various oxidases reductases which catalyze one-electron-transfer reactions, uncoupled endothelial nitric oxide synthase (eNOS) and inducible nitric oxide synthase (iNOS), lipoxygenase (LOX), and microsomal cytochrome P450 enzymatic metabolism of xenobiotic compounds [2]. As a consequence of the purine’s metabolism in the cytoplasm, carried out by xanthine oxidase (XO), there is also a production of another potent ROS [5].

A FR is an atom or molecule that has an unpaired electron in the external orbit, making it highly unstable and reactive. These species can be stabilized by capturing the needed electron from nearby biomolecules [6]. When these species are not eliminated by antioxidants, or are produced in excess, oxidative stress appears; thus, oxidative stress is a process characterized by a biochemical imbalance between FR or ROI and antioxidants [7]. Oxidative stress has implications for body homeostasis because FRs damage proteins, lipids, DNA, and organelles by reacting with them. It is directly linked to inflammation since inflammatory cells secrete a large number of cytokines and chemokines responsible for the production of new ROI and RNI in phagocytic and non-phagocytic cells through the activation of protein-kinases signaling [8], so we can say that oxidative stress is a cyclic process which has a positive feedback.

Biological systems have developed the ability to detoxify chemically-active ROS and RNS. These antioxidant mechanisms include complex non-enzymatic systems, such as glutathione (GSH), vitamins A, C and E, as well as enzymes such as catalase, superoxide dismutase (SOD), and various peroxidases. Insufficient levels of antioxidants, or inhibition of the antioxidant enzymes, also promote oxidative stress [9]. An important component of the detoxificant cellular system is the transcription factor that controls the expression of a large pool of antioxidant and cytoprotective genes in response to stimulation of oxidants or electrophilic molecules: This is the nuclear factor E2-related factor 2 (Nrf2). Nrf2 is negatively regulated by Kelch-likeECH-associated protein 1 (Keap1) [10].

## 2. Oxidative Stress, Inflammation and Chronic Diseases

As we discussed above, when an imbalance between FR and antioxidants, due to an uncontrolled and excessive generation of ROS takes place, oxidative stress occurs. These ROS, especially those derived from mitochondria, stimulate the activation of mediator signaling molecules as the transcription factor nuclear factor kappa-B (NF-κB) [11], that up-regulates the production of inflammatory cytokines, such as interleukin-1β (IL-1β) or tumor necrosis factor-α (TNF-α) [12] and others mediators, as iNOS or cyclooxygenase-2 (COX-2) [13]. Moreover, ROS can damage cellular lipids, lipid peroxidation products, and lipid-derived aldehydes as malondialdehyde (MDA), 4-hydroxy-2-nonenal (HNE), and acrolein, which are implicated in numerous oxidative stress-induced inflammatory diseases with harmful effects [14]. Proteins and DNA may also be damaged by ROS; the DNA damage can cause mutations and is implicated in the initiation and/or promotion of inflammation-mediated carcinogenesis [15]. NF-κB is activated by an important type of membrane receptor that is related to oxidative stress and inflammation, and whose regulation is the mechanism of action for certain antioxidant molecules to inhibit the inflammatory process. These membrane receptors are the toll-like receptor 4 (TLR-4) [16,17].

Inflammation is a normal protective response to a variety of cell and tissue damage. Its role is to remove harmful cells and tissues, as well as repair them. Uncontrolled inflammatory response results in extensive cell and tissue damage, giving rise to normal cell and tissue destruction, which is associated with chronic inflammation and various human chronic diseases [18]. ROS and inflammation have been identified in the pathogenesis and development of a great number of unrelated diseases including cancer [19]; nervous system diseases, such as psychiatric disorders [20], Alzheimer’s [21], Parkinson’s [22] and traumatic brain injury [23], cardiovascular diseases as atherosclerosis [24], metabolic diseases as diabetes mellitus II [25], periodontal disease [26], chronic kidney disease [27], gastrointestinal autoimmune diseases (Ulcerative colitis and Crohn) [28], other autoimmune diseases as multiple sclerosis [29], rheumatoid arthritis [30], hepatic diseases [31], and others as fibromyalgia [32,33].

Taking into account the recounted above, production of FR is an inevitable consequence of cellular respiration. If it is not controlled, it may lead to inflammatory and chronic diseases. Thus, what can we do? We can avoid an excessive production of these FR, or we can control antioxidant systems using free radical scavenger molecules such as melatonin (MLT), a potent antioxidant.

## 3. Melatonin: An Overview

**Figure 1:**
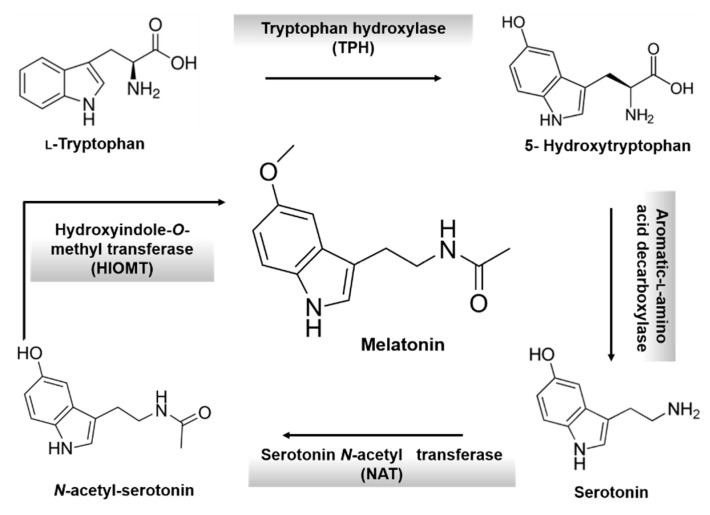
Biosynthesis of melatonin from tryptophan.

Melatonin is an endogenous hormone mainly synthesized and secreted by the pineal gland [34], which was isolated and chemically identified as *N*-acetyl-5-methoxytryptamine for the first time in the 1960’s [35]. Tryptophan is the precursor molecule for the synthesis of MLT (Figure 1). This essential amino acid is transformed to serotonin. Then, serotonin is converted into MLT in two sequential reactions involving two enzymes: serotonin-*N*-acetyl transferase (NAT) and hydroxyindole-*O*-methyl transferase (HIOMT) [36,37]. MLT is also produced by immune system cells, brain, airway epithelium, bone marrow, gut, ovary, testes, skin, and others [38]. MLT, and its derivatives’, behavior as free radical scavengers are well documented [39]. There is a large body of evidence of the use of MLT as a potent antioxidant because of such behavior. Below the potential use of MLT in various diseases given its antioxidant activity is described.

MLT possesses certain advantages when compared with other antioxidant molecules due to its important physical and chemical properties. It is a hormone found naturally in the body. The amphiphilicity of MLT enables molecules to enter all organs and subcellular compartments which represents a difference with other physiological radical scavengers. Furthermore, MLT is estimated to detoxify, via its cascade of reactions, up to 10 radicals, increasing the effective concentrations [39]. Compared with other antioxidant, MLT has shown to have equal or better efficacy in protecting tissues from oxidative injury (Vitamin C and E). Another inherent characteristic of MLT is the selectivity for mitochondrial membrane, which is not shared by other antioxidants and may be the most interesting advantage of the pineal hormone [40].

Regarding its toxicity, acute toxicity of MLT in animal and human studies is extremely low [41], with a very wide margin of safety [42]. There are no data about serious toxicological effects. Some side effects have been observed in clinical studies, most of them related to sleepiness and fatigue [43]. Another described side effect in a six-month, double-blind, crossover study, using 3 mg/day of MLT, was the alteration of semen quality in healthy men [44].

## 4. Role of Melatonin in Cancer

Carcinogenesis is a long, multistep, and often slow process that may start with the accumulation of FR leading to mutations in the genetic material of cells and thus the transformation of a normal cell into a cancerous cell, which reproduces without control, resulting in an imbalance between proliferation and cellular death [8]. Cellular mediators and cellular effectors of inflammation are important constituents of the local environment of tumors. Inflammation leads to tumor processes and, conversely, in some types of cancer, an oncogenic change induces an inflammatory response. These mediators and cellular effectors of inflammation have many tumor-promoting effects as proliferation and survival of malignant cells, angiogenesis, and metastasis promotion subvert adaptive immune responses and alter responses to hormones and chemotherapeutic agents [45].

Key molecules involved in cancer and inflammation that are the link for these two processes include transcription factors, as previously mentioned, NF-κB or signal transducers and activators of transcription 3 (STAT3), inflammatory cytokines such as IL-1β, IL-6, IL-23 and TNF-α.

NF-κB is a significant coordinator of innate immunity and inflammation. It is also an important endogenous tumor promoter [46]; this is a protein complex that controls DNA transcription by the following mechanism of action: NF-κB inactivated state is located in the cytosol and complexed with the inhibitory protein IKBα. Different extracellular signals, such as oxidative stress, cytokines, FR, UV irradiation, or bacterial and viral antigens, can activate the IKB kinase (IKK). This IKK is an enzyme that phosphorylates the IKBα, which results in ubiquitination, dissociation of IKBα from NF-κB, and degradation of IKBα by the proteasome. This dissociation has, as a result, the NF-κB activation and its translocation into the nucleus, where it binds to specific sequences of DNA called response elements (RE). The DNA/NF-κB complex recruits co-activators and RNA polymerase, which transcribes DNA into mRNA [47,48]. The mRNA is transcribed into a large number of the pro-inflammatory gene family, including cytokines (e.g., TNF-α, IL), chemokines, adhesion molecules, and inflammatory enzymes (e.g., iNOS, COX-2, 5-lipoxigenase) [49].

Another important transcription factor is the already mentioned Nrf2. There is accumulated evidence that Keap1/Nrf2 mutations or unbalanced regulation lead to over-expression or hyperactivation of Nrf2 and may participate in tumorigenesis and be involved in chemoresistance of both solid cancers and leukemias. In addition to protecting cells from ROS, Nrf2 seems to play a direct role in cell growth control and is related to apoptosis-regulating pathways. Moreover, Nrf2 activity is connected with oncogenic kinase pathways, structural proteins, hormonal regulation, other transcription factors, and epigenetic enzymes involved in the pathogenesis of various types of tumors [11].

IL-1 and IL-6 activate the JAK-STAT3 (Janus-activated kinase), SHP-2-Ras-ERK, and Pl3K-Akt pathways, through which induce cell proliferation, survival, EMT/invasion, metastasis, angiogenesis, and inflammation [50]. Angiogenesis is a crucial process involved in cancer and metastasis that allows new blood vessel formation from pre-existing vasculature during some physiological processes like embryogenesis, wound healing, and the reproductive cycle in adults. Vascular endothelial growth factor (VEGF) and NF-κB are important factors involved in solid tumor angiogenesis [51,52].

A family of proteins involved in tumor progression are the endothelins which include endothelin-1, 2 and 3 (ET-1, ET-2 and ET-3). They are involved in vasoconstriction, pain, inflammation, and cancer [53]. These participate specifically in cell proliferation of vascular and non-vascular cells [54]. High levels of ET-1, the predominant isoform, have been found in the plasma of patients with solid tumors [55]. Thus ET-1 is a tumor marker in colorectal, prostate, liver, breast, and ovarian cancers. In these tumor cells, this peptide promotes cell proliferation, angiogenesis, and metastasis and suppresses apoptosis [56]. The *edn-1* is the gene that encodes ET-1. The edn-1 is transcribed to mRNA, this mRNA is translated to prepoET-1 and then endothelin-converting enzyme (ECE-1) forms the biologically active peptide ET-1 [57].

MLT has been shown to inhibit cancer cell growth in numerous studies and through different mechanisms of action. In the following lines of this review the main ways that seem to be involved in tumor inhibition by MLT are described.

MLT inhibits edn-1 mRNA expression (the first step in ET-1 synthesis), ECE-1 protein expression and the release of ET-1 from colorectal cancer cells *in vitro*. The inhibition of edn-1 expression is due to an inactivation of FoxO1 and NF-κB transcription factors [58]. Pharmacologic concentrations of MLT inhibit angiogenesis by suppressing VEGF mRNA and VEGF protein induced by the hypoxia mimetic CoCl2 in all three human cancer cell lines. Decreased mRNA levels by VEGF were found to be associated with decreased hypoxia-inducible factor HIF-1a protein levels [59]. This is the most important regulator of VEGF [60]. Haddadi *et al.* [61] evaluated the effect of MLT on the modulation of *TNF-α* gene expression, and they found that the oral administration of MLT modulated the TNF-α overexpression.

High doses of MLT (1, 2 and 3 mM) have been shown to cause significant and progressive suppression of DNA synthesis in a cell line derived from a murine colon carcinoma [62]. Another mechanism whereby MLT inhibits cancer cell growth is the decrease in telomerase expression in MCF-7 tumor cells. Telomerase is an enzyme responsible for telomere elongation which is activated in most human cancers [63]. Moreover, once tumors are formed, MLT also seems to control their growth. In hepatoma cells, MLT inhibits, via membrane receptor-mediated processes, the uptake and metabolism of fatty acids, including linoleic acid (LA), and its conversion to the mitogenic signaling molecule 13-hydroxyoctadecadienoic acid (13-HODE) [64]. Some studies have shown that MLT has an antitumoral effect alone or in combination with others antitumoral agents, in animal or *in vitro* models of pancreatic tumors chemically-induced by different agents [65,66,67].

MLT may exhibit anticancer activity through several mechanisms, including antiproliferative, antioxidant, and immunostimulating effects. Some authors have conducted studies to test whether MLT could be useful in the treatment of human cancer. They tested MLT activity alone, or in combination with chemotherapeutic drugs, and compared to conventional anticancer therapies. Table 1 summarizes some clinical trials that have tested the potential role of MLT as an antitumoral or as adjuvant in cancer therapies.

**Table 1:** Clinical trials that have tested the potential role of MLT as an antitumoral or as adjuvant in cancer therapies.

Tumor	Treatment	Results and Conclusions	References
Metastatic non-small cell lung cancer	MLT + CisP + etoposide	Better tolerance to chemotherapy. Improve the efficacy of chemotherapy in terms of both survival and quality of life.	[68]
Metastatic cancer	MLT	Decline in VEGF secretion and control of the neoplasic growth.	[69]
Chronic lymphocytic leukemia	Cyclophosphamide + somastostatin + bromocriptine + retinoids + MLT + ACTH	Partial remission after 2 months and continued treatment. Patients hadn’t got disease recurrence. No toxicity.	[70]
Metastatic melanoma	MLT + IL-2 + Cisp	No Cisplatin-related neurotoxicity was observed. Effective and well tolerated treatment. Clinical efficacy at least comparable to that obtained with a first-line therapy of dacarbazine plus interferon-α.	[71]
Non-Hodgkin’s lymphomas (NHL)	Cyclophosphamide + somatostatin + bromocriptin + retinoids + MLT + ACTH	70% of participants had a partial response. 20% of participants had stable disease. 10% progressed on therapy. The combination was effective in treatment of low-grade NHL at advanced stage.	[72]
Advances solid neoplasms: non-small cell lung cancer (NSCLC) or gastrointestinal tumors	NSCLC: MLT + CisP + etoposide or gemcitabine; Colorectal cancer: MLT + OxiP + 5-FU or MLT + Etoposide or MLT + 5-FU + FA; Gastric cancer: MLT + CisP + Epirubicin + 5-FU + FA or MLT + 5-FU + FA	Regression rate achieved in MLT patients treated significantly higher than in those treated with chemotherapy alone and 2-year survival rate significantly higher in patients concomitantly treated with MLT.	[73]
Metastatic solid tumour: lung cancer, breast cancer, gastrointestinal tract neoplasms, head and neck cancers	Lung cancer: MLT + CisP + etoposide or MLT + gemcitabine; Breast cancer: MLT + Doxorubicin or MLT + Mitoxantrone or MLT + Paclitaxel; Gastrointestinal tumors: MLT + 5-FU + FA; Head and neck cancers: MLT + 5-FU + CisP	1-year survival rate and the objective tumour regression rate significantly higher in patients concomitantly treated with MLT than in those who received chemotherapy alone. MLT significantly reduced the frequency of thrombocytopenia, neurotoxicity, cardiotoxicity, stomatitis and asthenia.	[74]
Lymph node relapses due to malignant melanoma.	MLT	Disease-free survival in melanoma patients surgically treated for regional node recurrence was significantly higher in MLT-treated individuals than in controls.	[75]

Abbreviations: MLT, Melatonin; ACTH, Adrenocorticotropic hormone; CisP, Cisplatin; 5-FU, 5-Fluoracile; FA, Folates; OxiP, oxiplatin. In all cases MLT dose was 20 mg/day.

Results obtained from clinical trials suggest that MLT is an effective treatment for some types of neoplasm, either by improving the progression of the disease by controlling the growth and size of tumors, improving the effectiveness of classic chemotherapies or improving tolerance to them. Results also show improvements in the quality of life for patients when MLT is included in the anticancer therapy and, in some cases, significantly higher survival rates were obtained.

Finally, last years the protective effect of MLT against UV radiation has been proposed by its direct and indirect antioxidant activity (free radical scavenger and stimulation of antioxidant enzymes) [76]. UV radiation causes a neutrophilic inflammatory response. However, the main effect is related to tumor-initiating DNA mutations in melanocytes (melanoma) [77]. Several studies have demonstrated these effects [78,79].

## 5. Role of Melatonin in Oral Health

Oral health is essential to general health and quality of life, and according to the World Health Organization (WHO), is defined as the state of being free from mouth and facial pain, oral and throat cancer, oral infection and sores, periodontal (gum) disease, tooth decay, tooth loss, and other diseases and disorders that limit an individual’s capacity in biting, chewing, smiling, speaking, and psychosocial wellbeing.

Most prevalent inflammatory oral disease is gingivitis (gum tissue inflammation). Gingivitis, untreated due to a chronic exposure or accumulation of bacteria in the gingival sulcus of the mouth, will lead to an immune response where cells secrete lytic enzymes which, together with bacterial toxins, degrade collagen and glycosaminoglycans, This leads to a breakdown of bone and connective tissue that hold teeth in place. If not treated, the bones, gums, and tissue that support the teeth are destroyed [80,81]. Severe periodontal (gum) disease, periodontitis, may result in tooth loss. It is found in 15%–20% of middle-aged (35–44 years) adults [82].

**Figure 2:**
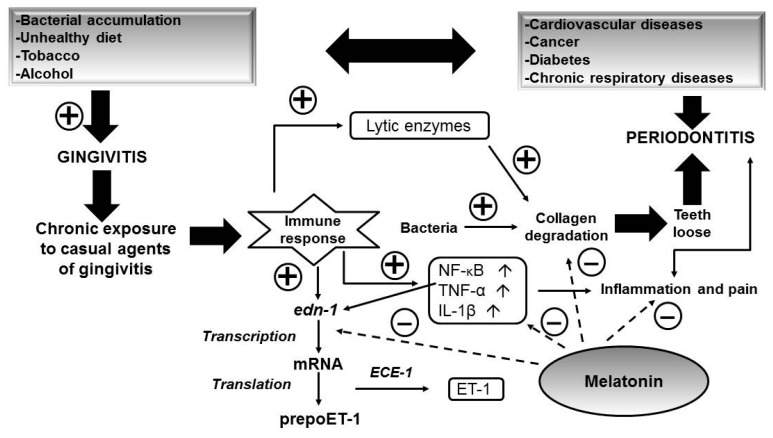
Diagram of the main mechanisms for the development of the oral diseases based on inflammation and oxidative stress: gingivitis and periodontitis and the role of MLT in their inhibition. Positive signs mean induction and negative inhibition. The line explains the cascade of reactions which relates gingivitis with periodontitis and involved exogenous and endogenous factors. The dotted line indicates where the activity of melatonin is targeted.

Risk factors for oral diseases include an unhealthy diet [83], tobacco use, and harmful alcohol use [84,85,86] (Figure 2). These are also risk factors for the four leading chronic diseases (cardiovascular diseases, cancer, chronic respiratory diseases, and diabetes) and oral diseases are often linked to chronic disease. Potentially, the most popular is the link between diabetes and periodontitis [87,88].

Eventually these diseases are linked to inflammatory processes and the mechanism of activation and propagation of the underlying inflammatory process is, in a great many cases, oxidative stress [89,90]. Avezov *et al.* summarizes the main sources of oxidative stress in the oral cavity and pathological outcomes [91].

Taking into account the direct relationship between the inflammatory process, oxidative stress, and these diseases, antioxidants are a possible mechanism in reducing gingival inflammation [92]. Thus, it can be thought that the establishment of antioxidant therapies, such as MLT, help to assist or remove the pathological complex network of interactions that may be established. There is already much evidence supporting this.

Some authors suggest that MLT saliva levels have a protective role in oral cavity tissue from oxidative stress damage [93,94,95]. Others propose that there is a direct relationship between these MLT levels and oral health [96,97,98].

In a rat pulp inflammation (pulpitis) model, TLR4/NF-κB signaling is activated [99,100] to increase serum levels of NF-κB, TNF-α, and IL-1β. MLT has been shown to suppress this increase and the percentage of necrosis of the injured pulp cells via TLR4/NF-κB signaling inhibition. MLT could also regulate the expression of TLR4/NF-κB signaling in LPS-stimulated human dental pulp cells [101]. In another experimental model of periodontitis in rats, MLT reduced inflammatory cytokine (IL-1b, TNF-α) levels, regulated oxidative stress parameters, such as malondialdehyde (MDA) or glutathione (GSH), and decreased periodontal tissue destruction [102]. The *in vitro* effect of MLT in gingival human fibroblasts has been also studied and it has been found that MLT has anti-fibrotic and anti-inflammatory properties via inhibition of endothelin-1 and TGF-B1, and increasing collagen and IL-10 levels [103].

Taking in to account that MLT is a nontoxic endogenous human hormone; it has powerful antioxidant, anti-inflammatory, and immunoenhancing activities, and participates in proliferation and bone remodeling [104]. It can be concluded that MLT is a potential candidate for the treatment of oral diseases.

## 6. Role of Melatonin in Kidney Chronic Disease

Chronic kidney disease (CKD) is characterized by increased levels of oxidative stress and inflammation. Oxidative stress and inflammation promote renal injury via damage to molecular components of the kidney by different mechanism of action. ROS oxidize the amino acids in the nephron, resulting in the loss of important functional properties [105,106,107], lipid peroxidation of cell membrane decrease membrane viability [108], and cleavage and crosslinking of renal DNA occurs leading harmful mutations [109,110]. Moreover, other ROS interactions in the nephron give rise to secondary radical production [111].

The therapeutic and protective effect of MLT against this kind of kidney damage has been shown in animal models [112,113]. There are a large number of studies of oxidative stress induced by exogenous drugs in which MLT has demonstrated a protective role; for example, in an oxidative stress animal model after a cisplatin administration [114].

Other kidney disease, which the use of MLT seems interesting, is ischemia/reperfusion injury (IRI), a major problem in clinical renal transplantation. Severe IRI of cadaveric kidney grafts, often caused by prolonged cold storage, contributes to delayed graft function and acute renal failure [115]. It is generally believed that ROS and RNS play the principal role in the mediation of IRI [116]. Li *et al.* have demonstrated that MLT protects kidney grafts from IRI through the inhibition of NF-κB and apoptosis after experimental kidney transplantation by improving recovery of renal and cellular function, reduces histological tubular damage index, and increases survival after reperfusion. As a pathophysiological explanation, MLT was shown to significantly reduce oxidative stress (lipid peroxidation) and induce SOD, while down-regulating NF-κB and iNOS and subsequently caspase-3-dependent apoptosis [117].

## 7. Role of Melatonin in Fibromyalgia

Fibromyalgia (FM) is a syndrome characterized by widespread pain, diffuse tenderness, and multiple symptoms including fatigue, sleep disturbances, cognitive dysfunction, and depressive episodes [118]. Since the main symptoms in FM (pain, stiffness and fatigue) are located in the muscles, muscle biopsies, mostly from the trapezius, have been studied. Biopsies of muscle have demonstrated inflammatory markers, subsarcolemmal mitochondrial accumulation, abnormal mitochondria, higher incidence of ragged red fibers, and defects of cytochrome-c-oxidase (complex IV of oxidative phosphorylation) [119,120]. Most recent studies have shown the implication of mitochondrial oxidative stress in the peripheral nociception described in FM as an important symptom which is mediated by inflammatory activation [32].

There is a phase II, randomized, double-dummy, controlled trial showing that the exogenous MLT administration (10 mg/day) increases the inhibitory endogenous pain-modulating system as assessed by the reduction on the numerical pain scale (0–10). In this study MLT alone, or associated with amitriptyline, was better than amitriptyline alone in improving pain on the visual analog scale [121]. Another randomized trial demonstrated that administration of MLT alone, or in combination with fluoxetine (3–5 mg/day), was effective in the treatment of fibromyalgia [122]. Taking into account the implication of oxidative stress in FM and the reported results, we believe that the mechanism of action implicated is the MLT activity as antioxidant.

## 8. Role of Melatonin in Inflammatory Bowel Disease

Chronic inflammation of intestinal tract or part thereof leads to the two major inflammatory bowel diseases (IBDs): Crohn’s disease (CD) and ulcerative colitis (UC) [123]. Although the main characteristic of both diseases is a relapsing immune activation which leads to an uncontrolled inflammation of the intestinal mucosa, they have different clinical symptoms and different histopathological characteristics [124,125]. UC is located at the large intestine (colon) [126] and CD can affect any part of the digestive tract from the mouth to the anus [127,128]. The role of ROS and RNS in the pathogenesis of both diseases [28,129] is known; that is, despite an exacerbated immune response in both diseases, each follows a different pathway to cause damage in intestinal cells. An excessive T helper 1 (TH1) response is linked with CD and an excessive TH2 phenotype is associated to the development of UC [123].

In UC patients significantly higher serum concentrations of cytokynes such an IL-13 (anti-inflammatory citokyne), IL-17 (proinflammatoty citokyne), C reactive protein (CRP) has been observed [130]. Other UC markers are IL-1β [131] and COX-2 [132]. Pentraxin 3 (PTX3) [133], which is not merely a marker, but directly involved in the proinflammatory process, as it facilitates neutrophil infiltration and elevates levels of proinflammatory cytokine as TNF-α [134,135].

The transcription factor NF-kB plays a fundamental role in the UC pathogenesis by regulating the expression of cytokines [136], as in carcinogenesis as we have seen above. Intercellular adhesion molecule-1 (*ICAM-1*) and *TNF-α* gene promoters have binding sites for NF-κB [137]. 8-oxo-7′8-dihydro-2′-deoxyguanosine (8-oxo-dG) in the rectal mucosa may be another useful biomarker for detecting patients with UC-associated neoplasia [138].

An important event in the UC is the increased intestinal permeability which occurs by a decline in expression of a tight junction protein (occluding) mediated by a Th2 cytokine: IL-13 [139]. This increased permeability, in the case of animal models of dextran sulfate sodium (DSS) induced colitis is accompanied by an increase in plasma levels of LPS [140], which is an indirect marker of gut bacteria in the systemic circulation [141].

It is known that there is a direct relationship between the UC and colon cancer; the duration of UC is proportional to the risk of suffering this kind of tumor [142]. The use of antioxidants in UC treatment may, therefore, prove interesting. Some authors have tested the role of MLT in this disease. Table 2 lists the obtained results and the impact caused by the administration of MLT in UC different models.

**Table 2:** Results and effects achieve with the administration of MLT in different animal models of induced UC by different chemical agents.

Study	Colitis Model	Results	Effects
Trivedi and Jena [143]	Dextran sulphate sodium (DSS) induced colitis	↓ Colon length observed in mice with UC	Increase intestinal surface
		↓ Levels of inflammatory markers: MPO, IL-17, IL-6, TNF-α, NF-κB, COX-2, STAT3	Anti-inflammatory at the systemic site
		↑ Nrf2, NQO-1 and GSH	↓ Oxidative stress involved in UC
		Antifibrotic effect: ↓ MMP-9 and CTGF	Decrease in the loss of function of intestinal tissue
		↓ 8-Oxo-de expression	↓ Oxidative DNA damage
		↓ Occluding expression	↓ Elevated gut permeability
		↓ LPS plasma levels	↓ Gut bacteria in the systemic circulation
Li *et al.* [144]	2,4,6-Trinitrobenzene sulfonic acid induced colitis	↓ mRNA levels for TNF-α and ICAM-1 colon tissues	↓ Colitis symptoms: rectal bleeding and occult blood and ↓ frequency and severity of mucosa damage dramatically
		↓ NF-κB-DNA translocation and mRNA expression by ↑ I*κ*Bα	↓ Expression of inflammatory cytokines
		↓ Inflammatory infiltrate of neutrophils, lymphocytes, and macrophages	↓ Severity of mucosa injury and alleviate colitis symptoms
Trivedi *et al.* [145]	1,2-Dimethylhydrazine dihydrochloride (DMH) and DSS induced colitis-associated colon carcinogenesis (CACC)	↓ Tumor multiplicity, significantly ↓ in number of aberrant and abnormal crypts in the colon	Ameliorative effect on the progression of colon carcinogenesis
		Significantly ↓ levels of inflammatory markers: MPO, IL-17, IL-6, TNF-α	Anti-inflammatory effect
		Significantly ↓ in NF-κB, COX-2, and STAT3 levels in the colon of mice with CACC	
		Significant ↓ TBARS and ↑ GSH levels in the colon	Antioxidant effect
		Significantly ↓ autophagy as revealed from the expression pattern of Beclin1, LC3-II/LC3-I ratio, and p62	↓ Autophagy in the colon of mice with CACC
		Significant ↑ Nrf2, NQO-1, and HO-1	↓ Oxidative stress
		↓ CACC-associated DNA damage as well as oxidative DNA damage in the colon of mice	Protective role in CACC
Tahan *et al.* [146]	Acetic acid-induced colitis	Significant ↓ TNF-α, IL-1β , IL-6, myeloperoxidase (MPO), and malondialdehyde (MDA) levels	Anti-inflammatory effect
		Significant ↑ GSH and SOD levels	Antioxidant effect
		Significant ↓ macro and microscopic lesion scores of the UC group	Protective role in UC
Sayyed *et al.* [147]	Acetic acid-induced colitis	↓ NF-κB inmuno histochemical expression	Anti-inflammatory effect
		↓ LP Levels	↓ Intestinal permeability
		↓ PTX3 Levels	↓ Neutrophil infiltration and proinflammatories Cytokines
Dong *et al.* [148]	Acetic acid or 2,4,6-trinitrobenzene sulfonic acid (TNBS) induced colitis	↓ Severity of gut injury and significantly ↓ of colon mucosal damage index (CMDI)	Protective role in UC
		Significantly ↓ of NO content and iNOS expression in colonic tissue	Antioxidant effect
		↓ PGE2 and expression of COX-2	Anti-inflammatory effect

UC, ulcerative colitis; MPO, myeloperoxidase; IL-17, interleukin 17; IL-6, interleukin 6; TNF-α, tumor necrosis factor-α; NF-κb, nuclear factor κ-B; COX-2, cyclooxygenase-2; STAT3, signal transducer and activator of transcription 3; NQO-1, NAD(P)H quinone oxidoreductase 1; GSH, glutathione; MMP-9, matrix metalloproteinase-9; CTGF, connective tissue growth factor; LPS, lipopolysaccharide; ICAM-1, intercellular adhesion molecule 1; CACC, colitis-associated colon carcinogenesis; TBARS, thiobarbituric acid reactive substances; HO-1, heme oxygenase 1; SOD, superoxide dismutase; LP, lipid peroxides; PTX3, Pentraxin 3; ↓, means reduction; ↑, means augmentation.

All these studies with animal models of UC show that MLT plays an important role improving the disease through multiple mechanisms of action, based on recognized antioxidant and anti-inflammatory capacity. Among other effects, MLT decreased tumor incidence, multiplicity, number of aberrant crypts per focus, and improved the histological profile or colonic lesions of mice with UC. MLT inhibited oxidant damage and inflammatory cytokines. MLT caused the decrease of colonic NO and PGE2 content, as well as the down-regulated expression of colonic iNOS and COX-2.

Besides experimental animal studies, there are data about MLT effects in human UC. Chojnaki *et al.* [149] carried out a clinical trial, in which four parameters were measured: Mayo Clinic disease activity index (MCDAI) (which took into account stool frequency, rectal bleeding, endoscopic findings, and physician’s global assessment), CPR plasma levels, hemoglobin plasma levels, and anxiety (according to Hamilton anxiety scale). Results, with a dose of 5 mg at bedtime, were positive for all parameters. This makes MLT to be a suitable candidate for the treatment of UC.

## 9. Role of Melatonin in Rheumatoid Arthritis

Rheumatoid arthritis (RA) is a systemic inflammatory disorder [150]. It is considered an autoimmune disease given the presence of autoantibodies as rheumatoid factor (RF) and anti-citrullinated protein antibodies (ACPA) [151,152]. These antibodies are involved in proinflammatory cytokine production *in vitro*, which in turn is known to be associated with systemic inflammation and RA [153]. As in other autoimmune and inflammatory diseases, the chronicity results in destruction of synovial joints due to continuous immune cell infiltration into the synovium [154]. RA is a disabling disease and has high rates of premature mortality [155].

Since RA is an inflammatory disease, key markers and molecules whose levels are increased and involved in RA pathogenesis are, among others, TLR3 and of IL-1β in CD34+ cells, CRP, fibrinogen [156], ROS, MnSOD mRNA, NOX2 mRNA [157], PTX3, which is related to other cardiovascular diseases [158], or anti-IgG4 hinge antibodies that may represent one mechanism of ACPA-mediated inflammation [159].

Oxidative stress plays a key role in RA. It has been reported that RA patients have higher antioxidant levels than healthy controls, but they are insufficient to prevent oxidative damage [160]. Some authors have demonstrated that there are increased levels of NO in RA patients’ serum, NO correlates significantly with disease activity, inflammatory markers and radiological joint status [161].

Given the profile of the disease, it is interesting to review what role MLT could play due to its antioxidant and anti-inflammatory activity. However, we found a double approach. Some authors defend the role of MLT as an adjuvant in the treatment of RA, but a substantial majority takes the view the MLT is implicated in the pathogenesis of the disease. Let us see the arguments for both.

Georges *et al.* reported that RA patients have higher nocturnal plasma levels of MLT than healthy controls. Additionally, they reported that MLT is present in the synovial fluid of RA patients and synovial macrophages have a specific binding site [162]. In another study we found strong indications about how MLT would be involved in modulation of immune responses by inducing the production and secretion of cytokines, such as IL-12 and NO production, too, by the involvement of synovial macrophages and human monocytic myeloid THP-1 cells. This leads us to believe that synovial arthritis’ symptomatology in RA may be related with ROS, produced by monocyte and macrophages, cytokines and diurnal rhythmicity of neuroendocrine pathways [163]. There are even authors who have evidence of the potential mediators in the role of MLT-mediated arthritis [164,165].

The arguments in favor are less numerous and are based on the antioxidant properties of this molecule. Forrest *et al.* used MLT as an adjuvant in RA therapy, but they did not find enough evidence of positive effects of MLT in RA [166]. Maestroni *et al.* discovered that MLT did not worsen RA progression [167].

It seems that it is becoming increasingly clear, the enhancing role of MLT in the pathogenesis of RA, contrary to what might be expected. Studies supporting this theory are more recent. As we have seen, there are theories about the mechanism of action involving the MLT in the pathogenesis of this disease; some point to those synovial macrophages responding to MLT with an increased cytokine production [168].

## 10. Conclusions

As we have seen throughout this review, MLT is involved in the inhibition of numerous pathways of numerous mediators of inflammation. They arise from oxidative stress or oxidative damage by both endogenous and exogenous molecules. Nowadays, in the age that antioxidants are in the spotlight as a possible solution to emerging diseases in the first world, which are directly related to oxidative stress and inflammation, MLT is in the running as a candidate in the form of monotherapy or as an adjuvant to other therapeutic agents. The only exception seems to be the pathogenesis of rheumatoid arthritis which must be more deeply investigated to elucidate the real mechanisms involving MLT.

Finally, note that the antioxidant and anti-inflammatory potential of MLT encourages us to study the possible use of this molecule, or its derivates, as an adjuvant in more aggressive therapies, or to minimize side effects associated with oxidative and inflammatory damage of certain medicines, which opens a very large field of study.
